# Evaluating the CYP-IAPT transformation of child and adolescent mental health services in Cambridgeshire, UK: a qualitative implementation study

**DOI:** 10.1186/s43058-020-00078-6

**Published:** 2020-10-14

**Authors:** Anne-Marie Burn, Maris Vainre, Ayla Humphrey, Emma Howarth

**Affiliations:** 1 Department of Psychiatry, University of Cambridge, Cambridge, UK; 2 MRC Cognition and Brain Sciences Unit, University of Cambridge, Cambridge, UK; 3 School of Psychology, University of East London, London, UK

**Keywords:** Child and adolescent mental health services, CYP-IAPT, Qualitative, Implementation, NPT, CFIR

## Abstract

**Background:**

The Children and Young People’s Improving Access to Psychological Therapies (CYP-IAPT) programme was introduced to transform Child and Adolescent Mental Health Services (CAMHS) across England. The programme comprised a set of principles that local CAMHS partnerships were expected to operationalise and embed with the aim of increasing access to services and improving the quality of care. This study explored how the implementation of the CYP-IAPT programme was executed and experienced by CAMHS professionals in the county of Cambridgeshire (UK), and the extent to which the CYP-IAPT principles were perceived to be successfully embedded into everyday practice.

**Methods:**

We analysed 275 documents relating to the CYP-IAPT programme issued between 2011 and 2015. We also conducted a thematic analysis of 20 qualitative interviews, undertaken at two time points, with professionals from three CAMHS teams in Cambridgeshire. Analysis was informed by implementation science frameworks.

**Results:**

Document analysis suggested that the CYP-IAPT programme was initially not clearly defined and lacked guidance on how to operationalise key programme principles and apply them in everyday practice. There was also a degree of programme evolution over time, which made it difficult for local stakeholders to understand the scope and aims of CYP-IAPT. Interviews with staff showed low coherent understanding of the programme, variable levels of investment among stakeholders and difficulties in collaborative working*.* Barriers and facilitators to programme implementation were identified at individual, service and strategic levels. These in turn impacted the local implementation efforts and sustainability of the programme in Cambridgeshire.

**Conclusions:**

We identified factors relating to programme design and national and local implementation planning, as well as features of inner and outer context, which impacted on the delivery and sustainability of the programme. These findings can be drawn upon to inform the development and delivery of other local and national quality improvement (QI) initiatives relating to children and young people’s mental health.

Contributions to the literature

- The CYP-IAPT programme was rolled out across England to transform child and adolescent mental health services, but relatively little is known about how the programme was executed and experienced at a local level.
- We found that the national programme was initially not well defined and lacked implementation guidance for local teams. Interviews with local stakeholders reflected this lack of clarity around the aims and objectives of the programme, as well as variable investment among staff.
- Barriers and facilitators to local implementation were identified, which offer potentially valuable insights for planning and implementing other QI programmes directed at young people’s mental health.

## Background

The Children and Young People’s Improving Access to Psychological Therapies (CYP-IAPT) quality improvement (QI) programme was initiated in 2011 to fulfil a new vision of Child and Adolescent Mental Health Services (CAMHS) in England [1]. The underlying rationale of the initiative was that system-wide change could be achieved through embedding key principles into routine practice. The foundational principles of the programme focused on improving *access* to services, increasing service user *participation,* raising *awareness* of mental health issues*,* improving quality of care through *evidence-based practice* (EBP) and enhancing *accountability* through use of routine outcome monitoring (ROMs) [2, 3].

Implementation of the CYP-IAPT programme was supported by the use of Quality Improvement Collaboratives (QIC), which brought together teams from multiple organisations to share knowledge and learn about best practice with the aim of improving service quality. This was facilitated at national and regional levels by linking higher education institutions with local partnerships (consisting of statutory, third sector service providers and commissioners) to form ‘Learning Collaboratives’ which would provide a mechanism for implementing new QI practices. It was expected that these inter-organisational partnerships would transform service provision in their local area by developing regional implementation plans in collaboration with service users to reflect local needs and priorities, and by training existing staff in several evidence-based therapies based on the national CYP-IAPT training curricula [4]. However, despite the QIC model being widely used in health care, the evidence for this approach is equivocal [5, 6]. Whilst some studies find that QICs confer some benefits to patient care and outcomes [7, 8], others find there is insufficient evidence that collaboratives improve outcomes or processes [6]. Arguably, for a QIC to be effective, it is vital to create cohesive networks to facilitate care coordination and involve key individuals who act as liaisons to connect disparate groups [9]. Furthermore, organisational readiness [10] and buy-in from senior management are perceived to be key determinants of success and sustainability of QICs [11–13].

Evaluation of the CYP-IAPT programme implementation has been limited; at a national level, a full evaluation of service and user outcomes was not possible due to large amounts of missing data [14]. Furthermore, there has been little evaluation of how the implementation of CYP-IAPT has been experienced and embedded at a local level. One exception is a rapid internal audit of 12 partnership sites conducted by programme developers [15], which highlighted a range of barriers and facilitators of service transformation including the individual characteristics of practitioners and the particular implementation process chosen by a local service.

Good process evaluation is needed to determine the effectiveness and sustainability of (any) programme implementation in real world settings [5, 16]. Furthermore, formal evaluation provides an opportunity to capture ‘how to’ knowledge that tends to be lost over time [17]. There is merit in undertaking a local evaluation because it provides an in-depth account of how the local context influenced implementation and is a way of retaining tacit knowledge from stakeholders who have been involved in multiple QI efforts. This information gives a measure of success or lack of and can aid replication and optimisation of the intervention [5].

There have been considerable efforts to describe the process of moving an intervention from the research environment or its pilot phase to widespread use [18]. Proctor et al. [19] presented a heuristic model to guide implementation research in the context of mental health services, which distinguishes between the evidence-based programme or practices, implementation strategies, and implementation, service, and client outcomes. According to this model, implementation outcomes relate to issues such as the feasibility, acceptability and reach of the programme being implemented and are distinct from service level outcomes such as effectiveness and safety, and client outcomes such as satisfaction.

The aim of the current study was to examine the process through which CYP-IAPT was implemented in an early pilot site: the Cambridgeshire and Peterborough Foundation Trust (CPFT) which is part of the English National Health Service (NHS). It explored the extent to which the CYP-IAPT principles have become embedded in everyday practice and identified perceived barriers and facilitators to local implementation. In line with Proctor’s model of implementation research, we sought to (i) describe the implementation strategies offered by programme developers and those developed and used by local implementers and (ii) evaluate implementation outcomes, specifically feasibility, fidelity, acceptability and sustainability. We used qualitative methods to explore stakeholder descriptions of the implementation process and associated outcomes drawing on normalisation process theory (NPT) [20–22] to explore the social processes underpinning implementation, and the Consolidated Framework for Implementation Research (CFIR) [23] to identify specific factors that impacted on the implementation process.

## Methods

We undertook a document analysis to describe the intended CYP-IAPT implementation model as articulated by programme developers and to describe the local activities and strategies used to embed the CYP-IAPT principles into routine practice in Cambridgeshire. We conducted interviews with CAMHS staff at two time points (early and late in the implementation process) to explore their views and experiences of the programme implementation. Reporting followed the Standards for Reporting Implementation Studies (StaRI) [24] and Consolidated Criteria for Reporting Qualitative Studies (COREQ) guidelines (additional file 1) [25].

### Data sources and sample

#### Document analysis

Documents relating to the implementation and delivery of the CYP-IAPT initiative between January 2011 and December 2015 were identified through targeted online searches and by contacting key personnel involved in the programme. The start date marks the beginning of the initiative in Cambridgeshire concurrent with Wave 1 of the national CYP-IAPT programme. A total of 609 published and unpublished documents were identified including reports, technical documents, web content, briefings, and meeting minutes. Of these, 334 were excluded because they were duplicates or outside of the date range. A total of 275 documents were included in the analysis (Table 1). Documents were categorised in Excel by year of publication, level of document (National, Collaborative and Partnership/Trust), issuer and document type.

**Table 1:** Summary of data sources

Data	Sources	Total
Documents	E.g. briefings, progress reports, guidance/policy documents, steering group minutes, agendas, memos	
	National	85
	Collaborative	64
	Partnership & Trust	126
Total documents		275
Interviews		
T1	**Implementation team** (2 clinician managers, 1 Clinical Psychologist*)	3
	Service manager*	1
	**Frontline staff** (1 Mental Health Practitioners*, 2 Family Therapists*)	3
T1 total		7
T2	**Implementation team** (1 Clinical Psychologist, 1 Data Manager, 1 Project Manager, 1 (Commissioner)	4
	**Service managers** (1 Team Manager, 1 Support Manager)	2
	**Frontline staff** (2 Clinical psychologists, 1 Child Wellbeing Practitioner, 3 Family Therapists, 1 Participation Coordinator)	7
T2 total		13
Total interviews		20

*Participants interviewed at both time points

#### Interviews

We conducted 20 in-depth semi-structured interviews with professionals from three CAMHS teams within the Cambridgeshire and Peterborough Foundation Trust. Interviews were carried out at two time points: early implementation stage (T1) and full implementation stage (T2). Participants at both time points were purposively sampled to ensure a range of stakeholders would be represented (implementation team, managers and frontline staff). Table 1 details the number of interviews with each type of stakeholder. Participants’ years in service ranged between 3 and 16 years (mean 10 years) and no participants dropped out of the study.

Interview schedules were informed by the NPT framework [20–22] and specifically tailored for each stakeholder group (e.g. additional file 2). Participants were invited for interview by email, and a snowball sampling approach was used to recruit additional participants. The majority of interviews were conducted face to face by one researcher (A-MB) in a clinical setting; three were telephone interviews. Participants were given an information sheet prior to interview and taken through the consent process. Interviews lasted about an hour, were audio recorded, transcribed verbatim and anonymised. Research and Development approval was granted from the Cambridgeshire and Peterborough Foundation Trust.

### Analysis

#### Documents

Documents were imported to NVivo V11 and the framework approach applied [26]. An initial framework was developed by the research team drawing on the research questions and key CYP-IAPT principles (accessibility, participation, awareness, EBP, accountability). Documents were systematically indexed and coded inductively and deductively by an experienced coder (MV). Framework matrices were produced by another member of the team (A-MB) which further validated the first round of coding and enabled the organisation, reduction and interpretation of the data along a 5-year timeline.

#### Interviews

Thematic analysis [27] of the interview data began with three members of the research team (A-MB, AH, EH) independently reading and becoming familiar with a subset of transcripts and identifying preliminary themes. The team met to review the inductive codes and to develop a working codeframe. All transcripts were coded in NVivo V12 by an experienced qualitative researcher (A-MB) and reviewed by the team to finalise overarching themes. We drew on NPT which is widely used to explore the processes by which an intervention becomes embedded into everyday practice [20–22]. Themes were mapped onto the four main NPT components: coherence (making sense of the intervention), cognitive participation (buy-in/investment), collective action (work done) and reflexive monitoring (appraisal of the benefits of the intervention).

Additionally, we drew on the CFIR [23] to identify the barriers and facilitators to implementation [28], [29]. We mapped inductive and deductive themes from combined interview and document data to the CFIR constructs. NPT and CFIR are both well-operationalised and have been used in conjunction to explore the implementation of QI initiatives [30].

## Results

We present our findings in four sections: (1) ‘CYP-IAPT implementation guidance’, (2) ‘Local implementation process’, (3) ‘Local stakeholders’ implementation experiences’ and (4) ‘Barriers and facilitators to implementation’.

### CYP-IAPT implementation guidance

Analysis of national and collaborative documents told the story of a programme that was evolving over time. Initially, there seemed to be a lack of clarity in the documents regarding the articulation of the core components. For example, an early document listed only four CYP-IAPT principles [31]; in another key document, the principles [32] were not clearly defined; and in a later document [33], six principles were listed. Across the national and collaborative documents, not all principles received equal attention. Whilst 62% of the thematic coding referred to EBP or ROMs, there were fewer references to the principles of participation (16%), access (4%) and awareness (1%).

Programme developers did not provide an implementation plan, and over the 5-year period, there was little practical guidance in the documents as to how to achieve the aims of the programme. However, this was addressed by a document published, more than 4 years into the programme, which gave an overview of practical steps to embedding the specific principles into practice [34]. The document followed the six stages of implementation proposed by Fixsen et al. [35]. It was the only comprehensive material relating to implementation that we could find.

The lack of specific guidance relating to implementation strategies seems to reflect a deliberate attempt by developers to avoid ‘implementing from the top’ [36]. Instead, Learning Collaboratives in five regions were established as the key implementation mechanism for QI [32], based on the assumption that local areas could learn from their neighbours with respect to moving key aspects of the programme into practice. There was an expectation that partnerships would share experience and competencies of implementation within and across Collaboratives. Additionally, it was envisaged that CYP-IAPT trainees would cascade EBP to colleagues in their service and act as ‘change agents’, thus accelerating the transformation and facilitating whole service culture change. However, this champion role was not formally specified and no guidance was given how to cascade knowledge or influence colleagues’ practices.

### Local implementation process

In the early exploration phase, local activity centred on reaching consensus about the programme’s aims and objectives. An Implementation Lead was appointed to facilitate communication between the local setting and the Collaborative, and a Steering Board established to address the infrastructure needed to capture data and the mechanisms needed to release staff for training. A CYP-IAPT project group was formed to oversee the day-to-day delivery of the programme.

Echoing the emphasis of the national documents, much of the initial local implementation activity focused on embedding the use of EBP and ROMs. A Data Manager worked with teams and individuals to ensure that the rationale for data collection was understood, and data were collected and used to inform practice. At full operation, activities for implementing EBP included interviewing staff for training, providing training on supervision and using ROMs in everyday practice. A high level of local activity increased children and young people’s engagement in and feedback about services, including the set-up of a user participation group. A Participation Coordinator was appointed and many activities were undertaken to make clinic environments less intimidating and young people were consulted in relation to patient facing information.

The analysis of documents across a 5-year period identified a number of challenges to longer term sustainability of the programme. The national vision of sharing data across the partnership did not fit with the local governance structures and by the second year of full implementation, the attendance at the Steering Board had waned. Importantly, there were ongoing difficulties with inputting clinical outcomes data into the information technology (IT) system which led to clinicians not using it and created a considerable time burden for the Data Manager who needed to input them manually. Three years into the project, as several people fulfilling key roles left, meetings and in-house training stopped and the Steering Board was disbanded.

### Local stakeholders’ implementation experiences

The interview data shows how the programme was understood and experienced by CAMHS professionals. The key themes were mapped onto the four main NPT constructs (coherence, cognitive participation, collective action and reflexive monitoring) (Table 2). Within each of the constructs, we describe how participants’ perceptions, attitudes and experiences changed over the course of implementation. Example quotes are labelled by interview time point (T1, T2) and type of participant. There was an alignment in the views of participants across the three CAMHS teams so findings were merged.

**Table 2:** NPT construct

NPT construct	Main themes
Coherence	Lack of clarity about the CYP-IAPT programme
Cognitive participation	Variable levels of stakeholder investment in CYP-IAPT
Collective action	Work and resources needed to implement CYP-IAPT
Reflexive monitoring	Evaluating and embedding CYP-IAPT

#### Theme 1: Lack of clarity about the CYP-IAPT programme (coherence)

At the early implementation stage, there was a lack of clarity about the scope and aims of the CYP-IAPT programme. Many staff found it difficult to differentiate the programme from other service transformation models or initiatives with similar names. Participants described how the programme was introduced at a time when the local service was in a state of flux and ‘upheaval’, and staff with many years of service expressed apathy and ‘change fatigue’ generated by multiple service change initiatives. At the time, concerns related to the data from outcome measures being used to appraise staff performance. Criticisms related to the lack of information about how CYP-IAPT could improve the service or what their role would be in delivering it.

I felt we weren’t at all well informed on it. We weren’t even told that was a task that we were meant to be doing until…I just learned a bit on the job. (T2, Frontline staff)

A common misconception early on was that CYP-IAPT would be a completely new service delivered by a separate specialist team rather than a system-wide service improvement, a view reinforced by the fact that only a limited number of clinicians were sent on the training courses.

Maybe there was a bit of a perception that it was previously a separate service rather than it being a general transformation and what everybody should be doing. (T2, Implementation Team)

In the follow-up interviews, there was a strong consensus that the core principles in the CYP-IAPT model (awareness, accessible, accountability, EBP, participation) had been given different levels of prominence during programme implementation.

…they [principles] haven’t all had the same impact as each other, haven’t had an equal impact. (T2, Service Manager)

**Fig. 1:**
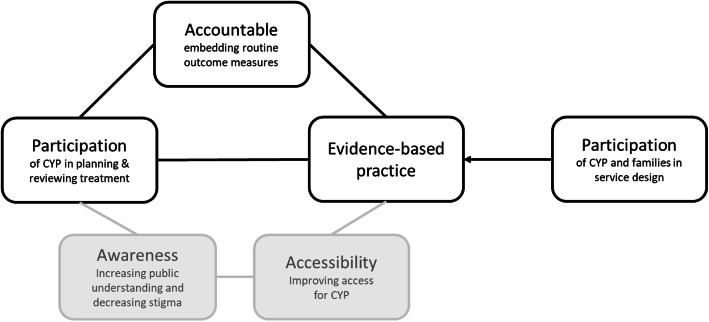
Depiction of participants’ perceived view of the CYP-IAPT principles at full implementation

The principle of *accountability* (i.e. embedding ROMs into everyday practice) was seen to be the defining principle of the programme, which was closely interconnected with the principle of *EBP* and the participation of young people in planning and reviewing their treatment (see Fig. 1)*.* However, the engagement of young people in the design of services was seen as a separate and unrelated activity from the overarching principle of *participation*. Meanwhile, as illustrated in Fig. 1, there was a general perception that the principles of *access* and *awareness* were not part of the core programme.

#### Theme 2: Variable levels of stakeholder investment in CYP-IAPT (cognitive participation)

A prominent feature of participants’ accounts was the variable levels of investment from key stakeholders which influenced local implementation efforts. Members of the implementation team had been highly invested in the programme and were credited for driving forward implementation plans. In particular, two champions (Data Manager and Participation Coordinator) were instrumental in embedding ROMs and engaging young people in the re-design of services. At that time, children and young people were more visible and their feedback gave rise to tangible improvements to the service.

…we had a very enthusiastic young service users’ Coordinator, and [name] was great and provided people to join interview panels. (T2, Frontline staff)

Variable levels of investment among frontline staff was largely attributed to the practitioner’s background. For some therapists, the principles ‘didn’t really fit with their philosophy’ and consequently they did not prioritise using them in practice. The general perception was that Psychologists were more invested in CYP-IAPT since the principles were more aligned with their training and skill sets.

…we were all clinicians. We all knew what the problems were and the language of CYP-IAPT in many ways spoke to us. (T2, Implementation Team)

Participants reported that a lack of buy-in from senior management constrained the local team’s abilities to initiate the organisational changes needed to deliver the project. Indeed, later stage interviews confirmed that senior managers stopped attending Steering Board meetings altogether where their input and authority was needed to make crucial decisions about funding and IT infrastructure. This contributed to a loss of momentum and gradually, CYP-IAPT disappeared from the agenda at local clinic meetings and the steering board eventually disbanded.

I think the steering boards, I know they were put on hold for a while because we couldn’t get attendance from senior management level to actually run them so we had to put them on hold really, which was really frustrating. (T2, Frontline staff)

#### Theme 3: Work and resources needed to implement CYP-IAPT (collective work)

A key part of the initiative was a training programme to enhance staff skills and knowledge to deliver evidence-based therapies. In the early implementation phase, the first cohort of trainees were highly critical of the training, reporting that courses had been rolled out in haste and were thus poorly prepared and disorganised.

My impression of the training process was that we were very much a guinea-pig year… I actually had a really poor experience of the training (T1, Frontline staff)

Perceptions of training improved over time with later trainees being more positive about the content and delivery and some reporting an increase in their confidence to deliver evidence-based treatments. Staff valued being given protected time to attend courses and the opportunity to learn from, and network with, practitioners from other teams.

I think what CYP-IAPT has done for me personally and professionally it’s given me lots of links and network opportunities with other services so that I can sort of talk to people about their practice and what they do in their teams and take that back to my team (T2, Frontline staff)

Although funding was provided to release staff for training, this created challenges for managers who were often required to fill training places at short notice, leaving the service understaffed in a climate of high service demand.

So you’d get an email to say that there was training and that did cause problems with staffing because we were pretty well down to the bones at the time. I remember we did grumble about that… I think sometimes we felt that our voices weren’t being heard. (T2, Service Manager)

A consistent theme was that the technological infrastructure to support the delivery of the programme was inadequate. Participants faced multiple technical problems collecting patient outcome data, and it took valuable time to carry out simple tasks. For example, they were unable to produce graphs which young people valued to visualise their progress and consequently many practitioners resorted to plotting graphs by hand. Another major obstacle was that the IT system was not aligned with other systems and this prohibited data from being automatically migrated to the national database, a requirement set for CYP-IAPT sites.

I mean this is just so typical of the NHS that things weren't joined up. So you have a separate data collecting system for CYP-IAPT to what was on offer for the rest of the service. And that's clunky. (T2, Implementation Team)

Attempts to resolve technical issues were counterproductive because the IT developers lacked an understanding of reporting clinical outcome data.

…we were still trying to convince the developer to embed the measures, and they really struggled with understanding clinical thresholds. So, they were producing graphs that were not really useful. (T2, Implementation Team)

A salient feature of participants’ accounts related to the collaborative work between different partnership organisations who were tasked with implementing the programme. Initially, it was seen as a positive to have a range of stakeholders ‘around the table’, but tensions arose due to conflicting organisational goals and priorities. Over time, it emerged that partner organisations (commissioners, statutory and third sector service providers) were not aligned in terms of their organisational structures, technical systems or working norms, and differences in the language used about mental health conditions and treatment were a barrier to effective communication. Despite the initially well-attended meetings, partner organisations were often represented by different people, which slowed progress and diffused responsibility.

…too many people were invited to the meetings, nobody felt they were a key part of it. Nobody kind of owned it, if you know what I mean, it was too dispersed. (T2, Frontline Staff)

#### Theme 4: Evaluating and embedding CYP-IAPT (reflexive monitoring)

Interviews explored whether the CYP-IAPT principles had become embedded into routine practice. An accepted view was that the principle of access had not been implemented locally due to concerns that ‘we would be flooded’ and it would overwhelm an already overstretched service. Nonetheless, there was a strong consensus that during the project, outcome measures had become embedded and were the ‘normal day to day way of doing things’ and participation activities had successfully placed young people at the forefront of shaping services. Although some staff had initially been sceptical about involving young people in service design, this view altered over time and staff later commented that the service increasingly recognised the value of service user feedback.

…it just really did feel at the time that young people were much more visible, not just a patient but a people who helped to shape what we were doing. (T2, Implementation Team)

The momentum of CYP-IAPT was difficult to sustain, and as noted above, gradually the programme stopped being discussed at team meetings. This was compounded by a high staff turnover; new staff joining the service were less aware of the principles, and consequently, some of the changes in practice began to disappear. Participants reported a lack of feedback from the national team to appraise whether local efforts had fulfilled the vision of the programme by improving services, and there were no clear metrics offered in order that teams could undertake this type of audit for themselves.

I think people would be quite interested to know where it is, six years on… there are always new agendas and priorities being set, some have a shelf-life and finish without anyone ever saying that they’ve finished…I think there’s a general sense about CYP-IAPT, that maybe that’s what might have happened. (T2, Manager)

### Barriers and facilitators to implementation

Using the CFIR [23] as a guide, we identified a number of factors from the combined document and interview data that influenced the implementation and sustainability of CYP-IAPT (summarised in Table 3) and described at strategic, service and individual levels.

**Table 3:** Barriers and facilitators to implementation of CYP-IAPT

Domain/element	Barriers	Facilitators
**Intervention characteristics**		
Evidence strength and quality Relative advantage		Many staff held the view that the CYP-IAPT programme was underpinned by evidence and provided a relative advantage over previous ways of working by standardising practice and widening the provision of evidence-based treatments
**Outer setting**		
Cosmopolitanism	Organisational differences and competing priorities within the CAMHS partnership impeded effective collaboration during local CYP-IAPT implementation	
**Inner setting**		
Compatibility	Some staff felt disenfranchised from a programme that was perceived to be at odds with their professional training and ethos	A key facilitator to CYP-IAPT implementation was highly invested staff. This was particularly the case where practitioners’ norms and values were compatible with the CYP-IAPT principles
Implementation climate	Programme was introduced at a time when the local climate was one of upheaval and change fatigue	
Readiness for implementation		
*Leadership engagement*	A lack of engagement from senior management in the Trust hindered local implementation efforts and prevented timely decisions being made at a higher level regarding much needed resources	The formally appointed local leadership team facilitated strategies and activities, and provided valuable links between CAMHS teams and other partnership organisations
*Available resources*	The limitations of the IT system were a barrier to practitioners recording and reporting outcome data and this reduced the quality of service evaluation and reflection on progress with young people First cohort’s impressions were that the training course was rolled out in haste. This negatively impacted their learning experiences and restricted access to knowledge and information Although national funding was a key facilitator for implementing CYP-IAPT, the funding was time limited and a lack of available resources meant backfill, training and dedicated staff roles could not continue. New staff did not benefit from training and were not familiar with the CYP-IAPT principles. Low staff capacity coupled with increasing service demands was a continual challenge for service managers.	Training was valued by trainees. Courses skilled up staff and embedded EBP and ROMs into everyday practice
*Access to knowledge and information*	At the start of the initiative, there was lack of readiness for implementation as staff felt they did not have sufficient information to understand the scope and aims of the initiative or to adequately understand their role in delivering it. A high turnover of staff led to a loss of skills and prevented knowledge continuity in the service.	
**Process**		
Champions	Dedicated staff moved on due to limited funding for their roles which impacted sustainability of the programme	Dedicated staff drove forward implementation and embedded EBP, ROMs and participation
Formally appointed internal implementation leaders	Local team lacked implementation expertise	
Reflecting and evaluating	A lack of feedback about measurable objectives or markers of success relating to CYP-IAPT made it difficult for staff to adequately reflect and evaluate overall progress of the initiative and it was unclear how the data was used or if at all, to drive service improvements.	

National funding was a key facilitator for implementing CYP IAPT; however, the funding was time limited and this meant backfill, training and dedicated staff roles could not continue. At a strategic level, there was no implementation strategy and therefore a lack of readiness for implementation; the training course was rolled out before it was ready, and staff had restricted access to information. Moreover, a lack of measurable objectives or markers of success relating to the programme made it difficult for local staff to adequately evaluate the initiative’s progress. It was also unclear how the data was used, if at all, to drive service improvements.

At a service level, the internal implementation leaders facilitated local strategies and activities and provided valuable links between CAMHS teams and other partnership organisations. However, a lack of engagement from senior management hindered local implementation efforts and access to resources. Specifically, lack of investment in the IT system was a restriction. Limited capacity to record and report outcome data reduced the quality of service evaluation.

A high turnover of staff led to a loss of skills and know-how. A lack of ongoing training meant new staff were not formally introduced to CYP-IAPT. This curtailed the extent to which the CYP-IAPT principles remained embedded in the service over time. Additionally, competing priorities between the partnership organisations were barriers to effective co-operation.

The key facilitator to CYP IAPT implementation was highly invested staff. Many held the view that the CYP-IAPT model provided a relative advantage over previous ways of working, although this was highly dependent on practitioners’ norms and values. Dedicated staff played an important role in championing ROMs and young people’s participation in service design. This influence was lost, however, when these members of staff moved on. The main challenge evidenced at an individual level, particularly in relation to embedding EBP, ROMs and participation, was the lack of adequate technical infrastructure and support.

## Discussion

Moving evidence-based practice into healthcare settings is a complex and challenging process [37–39]. The CYP-IAPT programme was introduced to change practices and embed a new way of working in CAMHS. Whilst the programme has been widely praised and integrated into subsequent mental health policy [40], limited evaluation at national and local levels means its success in driving service improvement and ultimately in improving mental health outcomes is unclear, and its value debated [36, 41, 42]. The purpose of this study was to explore how CYP-IAPT was delivered and experienced over time by local teams in Cambridgeshire including barriers and facilitators to implementation, with a view to informing decisions about how transformation efforts are sustained in mental health settings and how future QI initiatives should be delivered to maximise success. Our results, discussed in detail below, indicate that a lack of preparedness for implementation at national and local levels, a lack of senior management buy-in and difficulties with establishing an adequate IT infrastructure impacted on perceptions of acceptability, feasibility and sustainability. Moreover, differential emphasis by developers of the clinical principles comprising the programme contributed to a lack of fidelity, whereby only three of the five principles were implemented in practice.

### Lack of preparation for implementation at all levels

A fundamental problem was the lack of operationalisation of the implementation model by developers (see below), coupled with poor preparation and organisational readiness for change at a national level. As set out in numerous implementation frameworks [18, 35], these are all essential prerequisites for successful QI implementation. A sense that the programme was not quite ready for delivery emerged from both the document and interview analyses. The training programme was rolled out in haste and was not fully prepared for the first cohort of trainees (which included staff from Cambridgeshire), even though this could have been an effective way to establish engagement and a consistent understanding by staff. At the partnership level, the lack of senior management buy-in highlights the importance of properly laying the foundations to convince stakeholders that the programme will provide an effective solution to an existing problem [43]. In the preparation stage, there should be a ‘pre-emptive consideration’ of which stakeholder groups will (or will not) buy-in to the QI programme, so that conflicts can be resolved early on and align the needs and priorities of clinical staff and managers [44]. Furthermore, the poor IT infrastructure in Cambridgeshire was a major barrier to implementation and may be reflective of a bigger issue relating to a lack of early preparation at a national level to implement the programme. For instance, there appeared to be no overarching technical strategy to ensure systems could adequately collect data for effective monitoring of the programme.

Proper preparation requires investment in data collection and monitoring systems, yet the local system was not fit for purpose and thus created an extra burden for staff requiring them to put in high levels of effort to collect, analyse and report data. This is particularly important given the requirement that CAMHS submit outcome data to the NHS Digital Mental Health Services Data Set [45]. Currently, most clinicians use paper versions of outcome measures since organisational barriers to electronic collection, analysis and reporting still remain today.

### The programme was not clearly defined or understood

Our findings showed there was lack of clarity among local stakeholders regarding the scope of the programme, which is a crucial element for achieving successful implementation [46]. There was consensus that the programme was predominantly about embedding outcome measures and EBP into practice. This was reflected through national programme documents, which revealed that the principles were sometimes poorly articulated and to some extent differentially emphasised over time. This finding is resonant with other studies that have shown behaviour change interventions to be often poorly described [47] and therefore poorly understood [48]. A well-operationalised programme requires a clear description of the core components [49]. Research suggests there is a need to fully and precisely describe implementation strategies to ensure ‘reproducibility’ of the intervention and its effects [49]. Whilst the morphing and flexing of the programme appears to be a deliberate strategy to provide a flexible and phased approach to QI [36], it could be difficult to draw a boundary around what the programme actually was at any given time, and by extension difficult for partnerships to determine their progress in the context of poorly specified goals. This was addressed 3 years into the delivery of the programme via the publication of a document that could be used to guide transformation efforts [50].

### Lack of implementation guidance

There was a lack of practical and evidence-based implementation guidance to inform local teams as to how to embed the principles with everyday practice. The practice of providing implementers with an architecture (a plan of what needs to be transformed) rather than a blueprint (detailed plan of what and how) for transformation is consistent with a place-based approach to implementation, whereby differentiated models of service delivery will be required in different places in order to achieve equitable outcomes [51–54]. This model allows implementers the flexibility to use whatever strategies are locally appropriate, in order to achieve a stated goal, and avoids a top-down prescriptive approach which can undermine buy-in of local partners [55]. However, this approach assumes that those implementing the programme have knowledge of the range of effective strategies that could be used to implement change and the ways in which these can be tailored to local context. In Cambridgeshire, the local implementation team devised their own strategies which points to a lack of effectiveness of the QIC approach, which did not provide the local team with guidance and support as anticipated [5, 6, 56]. To some extent, this may have been because Cambridgeshire was part of the early pilot, but it highlights the need for early adopter sites to receive additional support and clear guidance regarding implementation strategies.

### Variable investment from practitioners

Low levels of programme coherence can lead to low cognitive participation and collective action [22] and change fatigue on the back of multiple transformations [57–60]. It was clear that there was high investment from practitioners whose therapeutic backgrounds (usually Psychologists) were perceived to be aligned with the ethos of CYP-IAPT; the previous internal audit conducted by programme developers found that embedding EBT was ‘easier for some disciplines than others’ [15]. Studies have shown that the quality of implementation can be enhanced when stakeholders value an intervention and perceive that changes will improve current practice [39, 61]. However, this runs the risk, as demonstrated here, of alienating some professionals who do not feel their therapeutic background aligns with the programme and is exacerbated when a training programme is only available for a subset of professionals. This can have the unintended consequences of some staff turning against a QI programme and creating a ‘them and us’ culture within a team [43, 62].

### The importance of key individuals and relationships

Consistent with the previous internal audit [15], our findings highlighted that the local implementation was facilitated by key individuals or champions. However, we found that their efforts were hampered by the lack of engagement from senior management, which impeded the mobilisation of essential resources. Progress stalled when key people left, and we found evidence that the CYP-IAPT principles were beginning to ‘wash out’ without continual focus on sustaining the programme. Collective focus and engagement of individuals at all levels is necessary for transformational changes to be sustained, as is succession planning to ensure turnover does not undermine progress [63, 64].

We also found that collaborative efforts between partnership organisations faced a number of challenges to implementation, which led to an over-reliance on individual members [9]. Although the collaboration of multiple agencies can facilitate implementation efforts, they also run the risk of problems such as ‘collaborative inertia’ [65], ‘free rider’ [66] and ‘social loafing’ effects [67]. Building relationships between different organisations requires time and effort, and maintaining focus can be challenging with shifting organisational priorities [43].

### Strengths and limitations

We used extensive database and lateral searches to identify all the documents issued between 2011 and 2015, and although we are confident in our search strategy, it is possible that we have missed some relevant documents. The experiences of the participants who were interviewed for this study may not be representative of others working in CAMHS within Cambridgeshire or elsewhere in the UK. We did not gather the views of members of the national team for crosschecking. The retrospective nature of this study means that we were unable to observe implementation activities as they occurred and therefore we have relied on the recollection provided by stakeholders. However, the interviews at Time 1 provided some verification of Time 2 accounts. We explored implementation outcomes as defined by Proctor et al. [19], although we did not gather objective data regarding programme fidelity or costs. Further, we were not able to evaluate the extent to which the initiative changed outcomes for services or patients. Notwithstanding, these findings are valuable for enhancing our understanding of the local context in order to inform other local transformation initiatives in young people’s mental health.

## Conclusions and implications for future transformation efforts

Despite efforts by local teams, we identified several barriers to implementation, which prevented the CYP-IAPT principles from being integrated into everyday practice. As indicated by quality improvement frameworks, before QI programmes are rolled out, time should be given to assessing readiness for change at both strategic and service levels and for preparing the organisational infrastructure to support the changes. This early stage of preparation is vital for laying the foundations necessary to engage stakeholders at all levels of the organisation. From the outset, there should be clarity about what the programme is and what it is trying to achieve, rather than a trickle of information over time with which it is difficult to keep up. The flexibility of a place-based approach to implementation is essential, but without training and support, it is unrealistic to expect local teams to have the requisite knowledge of evidence-based implementation strategies to do this. Early adopter sites, as Cambridgeshire was in this instance, are often in unchartered territory and may need extra support and training to undertake local implementation. A toolkit of implementation strategies along with support from an implementation expert could assist local teams to develop implementation strategies that are adapted to and reflective of local needs and priorities. The study findings are relevant for on-going national QI programmes directed at young people’s mental health interventions such as the mental health support teams into schools.

## Supplementary information

**Additional file 1.** COREQ checklist.

**Additional file 2.** Interview schedule.

## Data Availability

Consent was obtained from participants for the use of anonymised quotes in order to support the findings in this manuscript.

## Abbreviations

CAMHS: Child and Adolescent Mental Health Services
CFIR: Consolidated Framework for Implementation Research
CPFT: Cambridge and Peterborough Foundation Trust
CYP-IAPT: Children and Young People’s Improving Access to Psychological Therapies
EBP: Evidence-based practice
EBT: Evidence-based treatment
IT: Information Technology
NHS: National Health Service
NPT: Normalisation Process Theory
QIC: Quality Improvement Collaborative
ROMs: Routine Outcome Measures
