# Supplementary material for: Evaluating the CYP-IAPT transformation of child and adolescent mental health services in Cambridgeshire, UK: a qualitative implementation study
Source: Implement Sci Commun. 2020 Oct 14;1:89. doi: 10.1186/s43058-020-00078-6 (PMC7556968; doi:10.1186/s43058-020-00078-6)
Supplement: Supplementary file 1 — Additional file 1. COREQ checklist. [file 43058_2020_78_MOESM1_ESM.docx]

**COREQ check list CYP IAPT (Burn et al.,)**

| **Domain 1: Research team and reflexivity**  Interviewer/facilitator Credentials Occupation  Gender  Experience/training  Relationship to participants prior to study commencement?  Participant knowledge of interviewer  Interviewer characteristics | Dr Anne-Marie Burn (AMB)  PhD, CPsychol/Research Associate  Female  Academic researcher with qualitative expertise  Not reported in text but AMB had no relationship to participants prior to study commencement  Not reported in text but the participants were notified of the researchers’ reasons for carrying out the research in the study information sheet  Not reported |
| --- | --- |
| **Domain 2: Study Design**  Methodological orientation  Sampling Method of approach Sample size  Non-participation  Setting of data collection  Presence of non-participants  Description of sample  Interview guide  Repeat interviews  Audio/visual recording  Field notes  Duration  Data saturation  Transcripts returned | Pages 4-5  Sampling methods are detailed on page 4  None of the participants dropped out  Page 4  No  Pages 4-6  The interview guide is provided as a  supplementary file  We did not repeat interviews  Page 5  Fields notes were taken but are not reported  Page 5  The research team met and agreed at which point data saturation had been achieved.  Transcripts were not returned to participants |
| **Domain 3: Analysis and Findings**  Number of data coders  Description of the coding tree  Derivation of themes  Software  Participant checking  Quotations presented | Page 5  We have not included a coding tree but it is available on request  Page 5-6  Page 5  Participants did not provide feedback  Included in the results section pages 8-14 |
