# Supplementary material for: Evaluating the CYP-IAPT transformation of child and adolescent mental health services in Cambridgeshire, UK: a qualitative implementation study
Source: Implement Sci Commun. 2020 Oct 14;1:89. doi: 10.1186/s43058-020-00078-6 (PMC7556968; doi:10.1186/s43058-020-00078-6)
Supplement: Supplementary file 2 — Additional file 2. Interview schedule. [file 43058_2020_78_MOESM2_ESM.docx]

Interview schedule

| Frontline practitioners |
| --- |
| Can you tell me a bit about your role in CPFT?   - Current role - Years in service/year joined - Has your role changed over this time   Can you tell me a little a bit about your understanding of the CYP IAPT programme and your role in delivering it? |
|  |
| **If in post in 2011 Thinking back to 2011 when the programme was introduced…**  *-How well informed did you feel during the initial implementation period?*  *-What channels of communication were in place? How did you receive information about what was happening?*  *-do these continue to operate today?*  *-was there a process for communicating information back from the frontline to service managers and the implementation team?*  *-What helped people to communicate and work together during this early phase? E.g. meetings, information, presentations*  *-what could have been done differently? What would have helped make this better?*  **How committed were frontline practitioners to delivering CYP IAPT/specific aspects of the programme?**  **How well did frontline practitioners work together/with managers to implement the programme/specific aspects of the programme?**  **If in post after initial implementation phase**  **When you were first in post, how well informed did you feel about your role in delivering the programme/specific elements of the programme?**  *-what would have helped you to feel more informed? Probe communication/information/supervision*  **When you arrived in post, how well were people working together to deliver the programme/specific aspects of the programme?**  *-how well are people working together now?* |
| **In post in 2011**  **How well was the service working prior to the introduction of CYP IAPT?**  **What value did the specific CYP-IAPT programme or specific elements of the programme offer to the service?**  *-what benefits did you anticipate?*  *-Did you anticipate any negative consequences?*  **What did other colleagues think in terms of the programme’s value?**  **Were there some aspects of the programme that you or others thought might be particularly valuable/unhelpful?**  *-which elements?*  *-Why?*  *-is there anything that worked out better/worse that you first anticipated?*  **After initial implementation**  **What value (if any) does the CYP-IAPT programme or specific elements of the programme offer to the service?**  **What do other colleagues think in terms of the programme’s value?**  **Are there some aspects of the programme that are seen as particularly valuable or unhelpful?**  *-which elements?*  *-Any elements that seem redundant or unhelpful?* |
| **What changes were necessary in the structure of the organisation to deliver the programme/specific aspects of the programme? Probe about specific roles**  **- in your view** *are changes still needed to facilitate delivery?*  **What resources have been needed?**  *-are resources still needed? Human resource, IT*  **To what extent would you say that CPFT has been able to implement [**insert specific programme component**]**  **Are some of the elements of the programme more embedded than others?**  **-***Which elements*  *-why do you think this is?*  **Are there changes in organisational structures and roles that are still needed to ensure delivery of the programme?**  **Are there additional resources that are still required?** |
| **Now I’m going to ask some questions so that I can understand what work YOU DO NOW to implement specific aspects of the CYP IAPT programme**  ***Frontline practitioners***  *Involving CYP in the design and delivery of services*   - *Are CYP and families actively involved in making decisions about service delivery and design? Are you involved in that?* - *To what extent do you think involvement of CYP in the planning and delivery of services become a routine part of everyday practice* - *Are there any initiatives or activities in place to enhance the involvement of CYP in the design and delivery of services?* - *Is there any way that CYP can leave feedback about service provision?* - *What happens to the feedback? Are any changes made as a result?*   *Thinking about self-referrals;*   - *Are you involved in or aware of any initiatives/activity to increase access to CAMHS services?* - *To what extent are CYP and families able to access the service via self-referral?* - *How often do you see a patient who has self-referred?* - *Is the rate of self-referrals discussed at team meetings or in supervision?* - *To what extent do you think acceptance of self-referrals has become a routine part of everyday practice?*   *Thinking about working in partnership with CYP and families during treatment?*   - *Do you involve CYP and families in planning treatment decisions and goals?* - *How?* - *Is this actively encouraged by managers?* - *How?* - *What are the barriers?* - *To what extent do you think involvement of CYP in treatment decisions has become a routine part of everyday practice?*   *Thinking about the use of evidence based treatments*   - *What does evidence based practice mean to you?* - *Are you aware of those treatments that are endorsed by the CYP IAPT developers as evidence based?* - *To what extent is there a culture of evidence based practice in CPFT?* - *Which of these do you deliver?* - *How well equipped do you feel to deliver these treatments?* - *Have you received any training?* - *Are there any barriers to your use of EBTs?* - *Do you use any non EBT? When? Why?* - *Is your use of EBT monitored during supervision?* - *To what extent do you think EBT is a routine part of everyday practice* - *Are there any initiatives or activities in place to enhance the use of EBT?*   *Thinking about routine outcome measures*   - *In your opinion what is the purpose of routine outcome measurement?* - *How often do you use routine outcome measures in your everyday practice?* - *Which measures do you typically use and when?* - *Do you use ROMs to shape your practice with individual patients?* - *How? Do you find this useful?* - *If not, what prevents you from using the data in this way?* - *Is the data from ROMS used to give you feedback on your overall practice?* - *Is ROMS data used to guide service improvements?* - *How?* - *If not, why not?* - *To what extent do you think use of ROMs has become a routine part of everyday practice* - *Are there any initiatives or activities in place to enhance the use of ROMS?*   *Thinking about supervision*   - *How often do you receive supervision? From Whom? What is the function of these meetings? How helpful do you find it?* - *To what extent do you think clinical supervision is a part of everyday practice?* |
